# Tandem Substitutions in Somatic Hypermutation

**DOI:** 10.3389/fimmu.2021.807015

**Published:** 2022-01-07

**Authors:** Julieta H. Sepúlveda-Yáñez, Diego Alvarez Saravia, Bas Pilzecker, Pauline A. van Schouwenburg, Mirjam van den Burg, Hendrik Veelken, Marcelo A. Navarrete, Heinz Jacobs, Marvyn T. Koning

**Affiliations:** ^1^ Department of Hematology, Leiden University Medical Center, Leiden, Netherlands; ^2^ School of Medicine, University of Magallanes, Punta Arenas, Chile; ^3^ Department of Tumor Immunology, Radboud Institute for Molecular Life Sciences, Nijmegen, Netherlands; ^4^ Division of Tumor Biology and Immunology, Netherlands Cancer Institute, Amsterdam, Netherlands; ^5^ Department of Pediatrics, Leiden University Medical Center, Leiden, Netherlands

**Keywords:** apyrimidinic site, tandem dinucleotide substitutions (TDNS), strand slippage, tandem substitution, translesion synthesis (TLS), uracil-N-glycosylase (UNG)

## Abstract

Upon antigen recognition, activation-induced cytosine deaminase initiates affinity maturation of the B-cell receptor by somatic hypermutation (SHM) through error-prone DNA repair pathways. SHM typically creates single nucleotide substitutions, but tandem substitutions may also occur. We investigated incidence and sequence context of tandem substitutions by massive parallel sequencing of V(D)J repertoires in healthy human donors. Mutation patterns were congruent with SHM-derived single nucleotide mutations, delineating initiation of the tandem substitution by AID. Tandem substitutions comprised 5,7% of AID-induced mutations. The majority of tandem substitutions represents single nucleotide juxtalocations of directly adjacent sequences. These observations were confirmed in an independent cohort of healthy donors. We propose a model where tandem substitutions are predominantly generated by translesion synthesis across an apyramidinic site that is typically created by UNG. During replication, apyrimidinic sites transiently adapt an extruded configuration, causing skipping of the extruded base. Consequent strand decontraction leads to the juxtalocation, after which exonucleases repair the apyramidinic site and any directly adjacent mismatched base pairs. The mismatch repair pathway appears to account for the remainder of tandem substitutions. Tandem substitutions may enhance affinity maturation and expedite the adaptive immune response by overcoming amino acid codon degeneracies or mutating two adjacent amino acid residues simultaneously.

## Introduction

To effectively counter the virtually limitless possibilities of pathogen-derived immune challenges, B lymphocytes - representing a critical arm of the adaptive immune system - can generate a virtually limitless repertoire of structural B-cell antigen receptor (BCR) variants through somatic hypermutation (SHM) (1–7). Upon encountering an antigen, antigen-activated B cells initiate SHM, as well as class-switch recombination, through activity of activation-induced cytosine deaminase (AID) (8, 9).

AID deaminates cytosine (C) to uracil (U) preferentially in nucleotide motif WRCY (where W denotes A or T; R denotes A or G; and Y denotes C or T) on both DNA strands (10–14). This deamination locally instigates various substitutions through various mutagenic processing pathways (13–17).

When the U remains unmodified it will instruct a template T to all polymerases, resulting in C to T and G to A transitions. However, a U in the DNA is usually efficiently detected by uracil DNA glycosylase (UNG), which cleaves the base from the sugar-phosphate backbone, thereby generating a non-instructive apyrimidinic (AP) site. The AP site normally initiates faithful base excision repair (BER) involving an AP endonuclease (APE) and POLB (18, 19). During SHM however, the AP site can serve as a non-instructive template for the translesion synthesis (TLS) polymerase REV1, a dCMP transferase that can only insert a C opposite the newly generated AP site, thereby enabling C to G and G to C transversions (20–22). Alternatively, a single strand break at the AP site generated by APE allows POLH in complex with monoubiquitinated homotrimeric DNA clamp and replication processivity factor PCNA (PCNA-Ub) to access the site and generate about 8% of all A/T mutations by error-prone long-patch BER (6, 23–26).

Otherwise, the uracil is recognized as a U/G mismatch by the MSH2-MSH6 mismatch recognition complex and initiates the formation of a single-stranded gap flanking the U/G mismatch (17, 27). Upon binding of the MSH2-MSH6 complex, exonuclease 1 excises the uracil-containing strand to initiate non-canonical mismatch repair (ncMMR), where POLH in complex with PCNA-Ub generates the majority of A/T mutations (24, 26, 28–31).

Taken together, low fidelity translesion synthesis (TLS) DNA polymerases enable the generation of a large part of the spectrum of nucleotide substitutions. In these short- and long-patch ‘repair’ pathways, error-prone TLS DNA polymerases such as REV1, POLH, POLZ and perhaps POLI introduce mutations both at the position of the deaminated cytosine as well as in its near vicinity (28, 32–34).

In principle, all these mechanisms lead to single nucleotide substitutions (SNS). However, the occurrence of AID-instigated contiguous or ‘tandem’ substitutions, especially tandem dinucleotide substitutions (TDNS), has been described for several species. The term tandem substitution refers to contiguous substitutions resulting from a single DNA damage event, which could in theory lead to multiple discrete single nucleotide substitutions during its repair. The contribution of tandem substitutions to all AID-induced substitutions differs per species, ranging from 1,6% in mice (35) to nearly 60% in sharks (36, 37). Data on the frequency of this phenomenon from human *ex vivo* experiments in the immunoglobulin loci are currently lacking, but genome-wide the frequency of TDNS is estimated to range from 0,1% to 1% (38–41).

The biological consequences of tandem substitutions remain unknown. One advantage for TDNS is that they overcome the redundancy in the amino acid code in positions in the BCR where non-synonymous mutations are beneficial. This would in theory allow for faster repertoire diversification and hence more effective affinity maturation and humoral immunity (35, 42).

The molecular mechanism underlying the generation of tandem substitutions is debated. Evidently, a proportion of the observed contiguous substitutions are not, in fact, single event tandem substitutions, but multiple independent SNS occurring in contiguous nucleotides (43, 44). This may hold especially true in and around canonical AID hot spot motifs, where consequently SHM is most efficient (37). It appears however, that the incidence of tandem substitutions is far higher than expected from clustered SNS alone, indicating the existence of a mechanism creating contiguous substitutions in a single event (35).

Despite its potentially substantial role in the adaptive immune response, the mechanisms responsible for tandem substitutions and their relative contribution to the process of SHM has not been investigated in humans since the introduction of massive parallel sequencing. Here, we investigated the incidence of tandem substitutions in human peripheral blood B cells (PBMC) in healthy donors and patients with DNA repair deficiency and analysed their substitution motifs. We aimed to confirm association of previously implicated DNA repair mechanisms involved in the creation of tandem substitutions, as well as identify other potential mechanisms. Additionally, we investigated whether tandem substitutions indeed overcome amino acid redundancy by analysing the topographical distribution of TDNS in the V allele.

## Materials and Methods

### Sample Collection and Preparation

Peripheral blood samples were obtained with written informed consent from twelve healthy stem cell donors in compliance with the biobanking regulations of Leiden University Medical Center. Mononuclear cells were isolated by Ficoll separation and cryopreserved in aliquots. B cells were purified from aliquots of thawed cells by removal of non-B cells with magnetic beads (B cell isolation kit II; Miltenyi Biotec, Leiden, The Netherlands), routinely yielding a purity of >99% CD19^+^ B cells as assessed by flow cytometry.

### V(D)J Library Generation

Aliquots of 2x10^6^ B cells were processed according to the ARTISAN PCR protocol for unbiased amplification of BCR repertoires, which has very low amplification and sequencing error rates of 0.126 × 10^−3^ per bp (45). Full-length IgM and IgG VDJ were amplified from all twelve donors, while IgA and IgE VDJ. VJ-kappa and VJ-lambda were amplified from six donors each (46). Libraries were barcoded, pooled and amplified as single molecules in rolling circles on a total of fourteen SMRT cells on the RSII system (Pacific Biosciences, Menlo Park, CA, USA). Output sequence files were filtered with SMRT portal software for a minimum of eight sequencing passes. All sequences were annotated by IMGT HighV-QUEST (47).

Additional datasets of healthy donors, as well as UNG-deficient and MSH2- and MSH6-deficient patients were obtained from publicly available sources (48, 49) (**Supplementary Table S1**). Its amplification and sequencing methodology was previously validated and shown to be reliable for the identification VDJ of naïve and memory B cells alike (50).

### Sequence Selection

Sequences with identical V, D (if applicable), and J genes, identical CDR3 length and ≥95% pairwise identity in the nucleotide CDR3 sequence were analysed as a single sequence. Clonal expansions were reduced to the least mutated sequence to minimise the presence of amplification or sequencing errors. For the Leiden cohort healthy donors, sequences with no mutations, as well as sequences with >5% mutations in their V region were excluded from further analysis to minimize chance occurrences of consecutive mutated nucleotides whilst maintaining a reasonable number of mutations per sequence. Such selection of oligomutated sequences could not be performed for the publicly available datasets, as these datasets contained solely (often highly mutated) IgG and IgA rearrangements which, upon equally stringent selection, would have led to greatly reduced library sizes, precluding any meaningful analysis (46). From the publicly available data, only sequences containing one of the 50 most commonly rearranged IGHV alleles were considered, offering the option to completely perform *in silico* correction of these sequences.

For fair comparison, sequences with >5% mutations in the Leiden cohort were grouped in bins of 5-10% mutations, 10-20% mutations and >20% mutations and analysed identically to the less mutated sequences (**Supplemental Data**). Because the main findings in these data sets in essence corresponded to the findings in the less mutated sequences, yet were more susceptible to methodological imperfections such as less accurate ‘false tandem’ correction (due to lower ratios of observed:simulated TDNS, see below), these sequences were not incorporated in the main analysis.

### Identification of Mutations

We identified all SNS, TDNS, and longer contiguous substitutions in the V region of the 50 most commonly used IGHV alleles, 25 most commonly used IGKV alleles, and 25 most commonly used IGLV alleles, IGLV9-49*01 or IGLV10-54*01 (the latter two to have all IGLV families represented). We identified the germline nucleotide(s), mutated nucleotide(s), context (1 nucleotide on either side of the substitution), and their position within the V allele using Geneious software v10.1.3 (Biomatters Ltd., Auckland, New Zealand). Insertions and deletions were disregarded in this analysis.

The similarity analysis between *TDNS* and the Doublet Base Substitution (DBS) Signatures deposited in the COSMIC catalog (v3.1 - June 2020) (44) was measured by cosine distance using R (51).

### Predicted Frequency of Contiguous Substitutions From Multiple Mutation Events

The contribution of contiguous substitutions resulting from two or more independently occurring adjacent SNS, was modelled *in silico*. To have adequate numbers of control sequences, only the 20 most abundant V alleles were used for modelling.

Considering the observed mutation frequency of SNS per position and the distribution of particular substitutions per position, germline sequences were mutated *in silico* to a total number of mutations matching the mutation load of sequences observed in the *in vivo* data. These calculations were performed 100.000 times each for all rearrangements. Output sequences were analysed for single and contiguous substitutions, SNS and TDNS motifs and nonsense mutations. Any mutation clusters thus observed were considered to represent ‘false’ tandem substitutions and were subtracted from the actually observed frequency of tandem substitutions to obtain the true frequency of tandem substitutions in the dataset. Calculations for the complete dataset were extrapolated from this majority subset.

For the detailed modelling strategy, see: https://github.com/CATGUMAG/tandem-substitution-simulation.

The CMMRD patient repertoires mostly lacked IgM and both the patient and Rotterdam healthy donor repertoires were sequenced using a different approach and sequencing platform than the Leiden healthy donor cohort, which resulted in higher mutation loads and more false TDNS. Therefore, we did not pool these repertoires in our *in silico* mutation algorithm, but processed them separately and then independently predicted SNS mutation clusters as described for the Leiden cohort.

The paucity of unique sequences in the UNG-deficient patient’s library precluded detailed *in silico* correction as performed for the other datasets. To obtain the best approximation of corrected tandem substitution incidence in this dataset, *in silico* predictions were performed on pooled sequences of the IGHV3 and IGHV4 family.

### Mutational Resistance Scoring

A score was assigned to each position in IGHV, IGKV or IGLV to represent the chance that any random single nucleotide substitution will cause a synonymous mutation. For each codon in the human amino acid code, all 9 possible substitutions were classified as synonymous, non-synonymous or nonsense mutations. Since nonsense mutations would normally cause the BCR to be deleted from the repertoire and therefore not be represented in this dataset, these substitutions were disregarded and the chance of a random substitution leading to an amino acid change was calculated from the remaining options. Consecutively, for each position in the V region, proportional germline codon usage was calculated and multiplied by the chance that a codon would incur a synonymous mutation upon any random substitution. The results of all codons per position were added together to obtain the repertoire-broad chance of synonymous mutations per position (**Supplementary Table S2**).

This theoretically expected proportion of synonymous mutations was compared with the observed proportion of synonymous single nucleotide substitutions. When the observed frequency of synonymous mutations is higher than expected, this might indicate that BCR with non-synonymous mutations in this position have undergone negative selection and are no longer found in the repertoire. Conversely, lower observed synonymous mutation rates than expected may indicate a selective advantage for BCR altering their sequence in this position. However, it should be noted that this method assumes random substitutions across the V region whereas in reality mutations preferentially cluster around AID motifs and some substitutions occur preferentially over others (52). This method is therefore more suited for analysis of V region-wide topographical distribution of mutations rather than scrutiny of a single position.

After calculating the permissiveness of every position to incur non-synonymous mutations, the number of TDNS per position was counted and compared to the pattern of SNS to assess whether tandem substitutions are distributed randomly or preferentially cluster in certain areas of the V region.

We applied a Quasi-Poisson regression to establish if the number of tandem substitutions for each position in the V allele is correlated with the mutational resistance score. The analysis was performed using the R Stats Package (51).

## Results

### 6% of Substitutions Are Tandems

Twelve healthy donor peripheral blood B-cell receptor repertoires were sequenced using full-length, unbiased, massive parallel V(D)J sequencing. Selection of unique, clonally unrelated, antigen-experienced sequences carrying up to 5% mutations yielded 13.532 VDJ, 7.952 VJ-kappa and 7.598 VJ-lambda. Comparison to the closest germline allele allowed for identification of a total of 122.878 single nucleotide substitutions (SNS), 10.735 tandem dinucleotide substitutions (TDNS) and 2.615 longer contiguous substitutions. The longest contiguous substitutions were 8 nucleotides in length (**Supplementary Table S3**).

**Figure 1:**
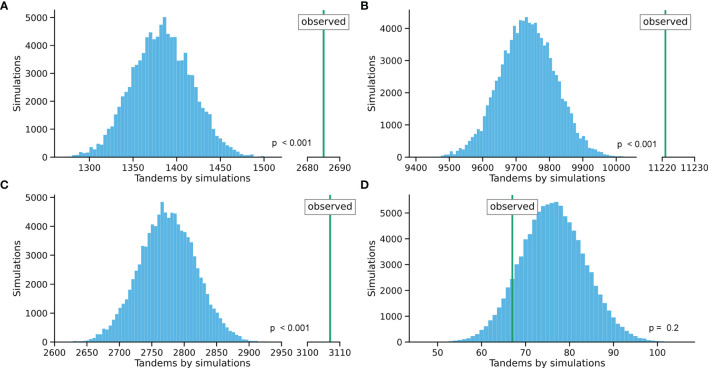
Comparison between the tandems found in the simulated datasets (distribution of 100,000 simulations) and observed values for each cohort. **(A)** Leiden healthy cohort (IGHV). **(B)** Rotterdam healthy cohort (IGHV). **(C)** Rotterdam MSH2/6 deficiency cohort (IGHV). **(D)** Rotterdam UNG deficiency cohort (IGHV). The distance between the distribution and the observed value indicates the number of tandem mutations after correction. Z-score analysis was performed to compare distributions.

Since mutation clusters of independently generated, but adjacent SNS are indistinguishable from single event tandem substitutions, we calculated the number of expected clustered SNS for each individual repertoire through *in silico* modeling (IGHV **Figure 1**; IGKV and IGLV and pooled IGV **Supplementary Figure S1**; **Supplemental Data 1.1-1.23**). Using the 20 most abundantly observed IGV alleles, synthetic immunoglobulin repertoires of matching size and V allele usage distribution were mutated 100.000 times *in silico*. These *in silico* repertoires were designed to exactly match the mutation rate for each nucleotide position in the sequenced repertoire. Since all mutations within this model were introduced in single steps, observed mutation clusters in the modelled data were annotated as ´false’ tandem substitutions.

Predicted ‘false’ tandem substitutions were subtracted from the total number of observed mutation clusters to obtain the frequency of single event tandem substitutions. Of all TDNS in the *in silico* simulated subset, 46,2% were predicted to represent two adjacent SNS generated in independent events. Extrapolating to the whole dataset (and counting the ‘false’ tandem substitutions as multiple SNS each), we observed 133.577 SNS and 5.775 TDNS. Therefore, the incidence of true, single event TDNS in human BCR *in vivo* was found to be 4,10%. Similar calculations for trinucleotide, tetranucleotide and pentanucleotide contiguous substitutions respectively showed incidence rates of 1,18%, 0,31% and 0,12%, containing 12,5%, 3,0% and 1,0% false positives, respectively. Cumulatively, 5,7% of all substitutions in human BCR were single event tandem substitutions, making their incidence rate much higher than observed in mice and in genomic human sequences outside the immunoglobulin loci (**Supplementary Table S4**).

### The Distribution of TDNS Matches SNS

**Figure 2:**
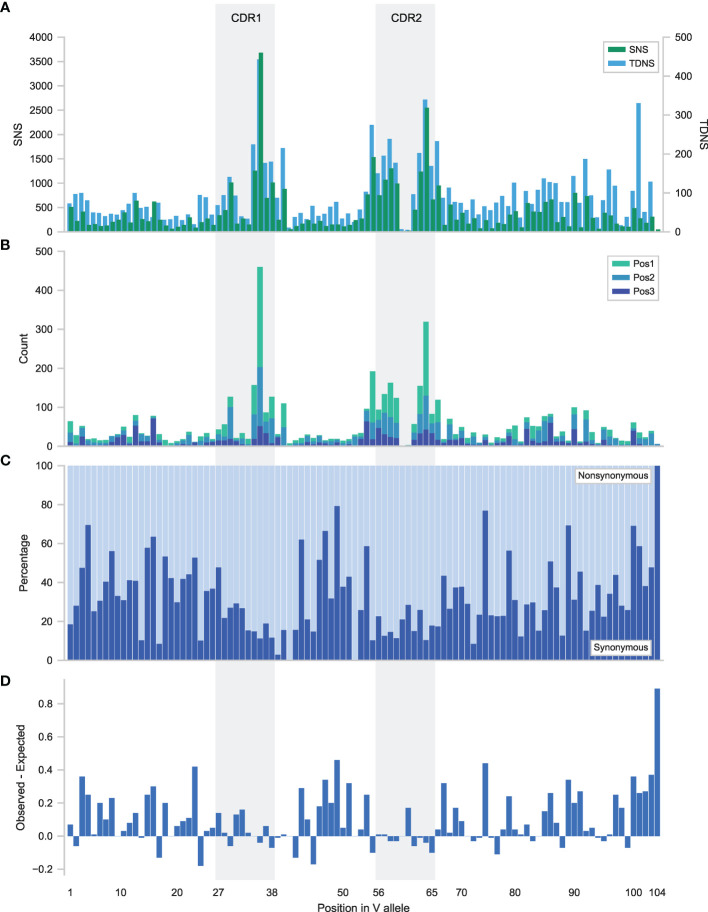
Distribution of tandem dinucleotide substitutions in the IGHV allele. **(A)** Distribution of single nucleotide substitutions (SNS) and tandem dinucleotide substitutions (TDNS) in the V allele. Amino acid positions 30-32 and 60-61 in CDR1 and CDR2 respectively, are present in only a small minority of V alleles and therefore mutations in these positions are rare events when considering the whole sequence library. **(B)** Nucleotide specific context within a codon. Relative contributions of tandem dinucleotide substitutions for VDJ. Results are split per position in the codon. Position 1 represents 5’ and middle base on the coding strand, position 2 represents middle and 3’ base and position 3 represents the 3’ base of the codon and the 5’ base of the downstream codon. **(C)** Proportion of synonymous and nonsynonymous mutations per position in the V allele. **(D)** Mutational resistance score. Plotted are the expected minus the observed frequencies of nonsynonymous mutations per codon position in the V allele. Increasingly higher values represent residues that are more resistant to mutation.

The distribution of all SNS and TDNS occurring in the most abundantly rearranged V alleles was mapped geographically up to IMGT amino acid position C104. As expected, mutation frequencies in complementarity determining regions (CDR) exceeded those of framework regions (FR).Specifically, structurally essential FR residues such as C23, W41 and C104 were strikingly less mutated than surrounding residues. Overall, the distribution of TDNS resembled those of SNS, indicating that mechanisms governing the generation and selection of TDNS are closely related to those of SNS (**Figure 2**, **Supplementary Figure S2A** and **Supplementary Figure S3A**). Also, the SNS follow an equal distribution pattern of mutations (Ts/Tv: 1,05) corresponding to previously described datasets (53) (**Supplementary Table S5A**). Despite their overall similarities, the relative abundance of TDNS differed from SNS in a number of positions. TDNS were overrepresented in a number of FR residues in the IGLV dataset (**Figure 2**, **Supplementary Figure S2B** and **Supplementary Figure S3B**). More commonly, however, FR residues contained fewer TDNS than expected. This relative scarcity of TDNS was most profound around structurally essential FR residues, where the observed SNS generally encoded for synonymous mutations (**Figure 2**, **Supplementary Figure S2C** and **Supplementary Figure S3C**). We hypothesized that TDNS in FR would be selected against, given how their higher potential for non-synonymous/replacement mutations (see below) would easily lead to deleterious effects on BCR integrity.

To test this hypothesis, we predicted the proportion of synonymous mutations at each position (Materials and Methods) and compared this to the observed proportion of synonymous mutations at that position. Subtracting the former from the latter resulted in a mutational resistance score, where progressively higher scores represented positions with more synonymous mutations than expected. Putatively, positions with high mutational resistance scores result from negative selection of BCR with non-synonymous mutations in these positions.

Indeed, we observed relatively few TDNS in positions with higher mutational resistance scores and we confirmed a negative selection of tandem substitutions in these structurally important positions using a Quasi-Poisson regression (**Figure 2**, **Supplementary Figures S2D**, **S3D**, **Supplementary Table S6**, p-value<0,05).

### Tandem Mutations Overcome Codon Redundancies

As more nucleotides are mutated at once, the chance of non-synonymous, i.e. amino acid replacement mutations, increases. To determine how often this occurred, TDNS were assigned to three groups according to their position in the codon: position 1 for substitutions of the 5’ base in the coding strand and the middle base, position 2 for the middle and the coding strand 3’ base, and position 3 for the 3’ base of one codon and the 5’ base of the subsequent codon. Substitutions in position 2 may only cause non-synonymous or nonsense mutations, whilst substitutions in position 1 and 3 may rarely cause synonymous mutations. Overall, 98,6% of TDNS encoded for at least one amino acid replacement, while only 71,6% of SNS encoded a non-synonymous mutation. Apparently, TDNS have the potential to expedite amino acid changes and thereby are likely to enhance adaptive immune responses.

**Figure 3:**
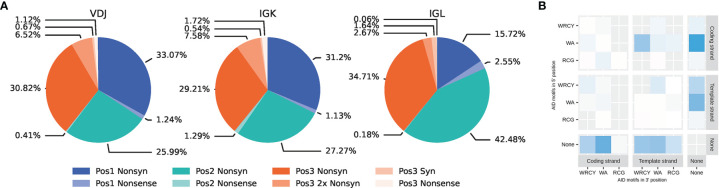
Relative contributions of synonymous, non-synonymous and nonsense tandem dinucleotide substitutions for VDJ, VJ-kappa and VJ-lambda. **(A)** Results are split per position in the codon. Position 1 represents 5’ and middle base on the coding strand, position 2 represents middle and 3’ base and position 3 represents the 3’ base of the codon and the 5’ base of the downstream codon. Tandem dinucleotide substitutions causing two separate synonymous mutations in position 3 are displayed as a separate category. **(B)** Analysis of AID motifs present in tandem substitution for IGHV sequences. The 3 motifs associated with AID activity (WRCY, WA, and RCG) were identified in position 5’ or 3’ of the tandem. The motifs were considered in forward (coding) and reverse (template) direction.

Additionally, TDNS in position 3 may potentially mutate two adjacent residues simultaneously, which was observed in 18.5% of such position 3-situated TDNS. It appears that this potential for accelerated affinity maturation comes at a price, because position 3-situated TDNS were less abundant than the other positions, supposedly due to negative selection of structurally unsound BCR or nonsense mutations. An exception was found in the IGLV library, where some of the previously described hotspots were situated in position 3 and compensated for the lower abundancy overall (**Figure 3**). Whether such relative scarcity of position 3 TDNS indeed resulted from increased negative selection pressure, could not be tested in this reverse immunology investigation.

### Most Tandem Mutations Are Juxtalocations

**Figure 4:**
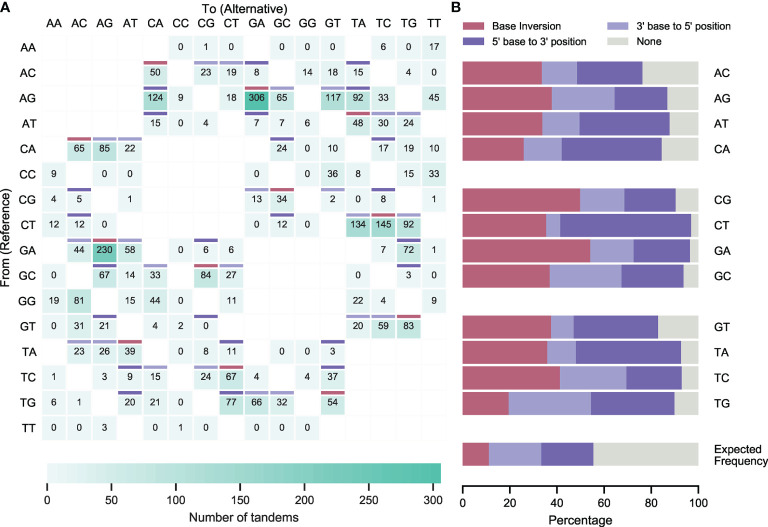
Corrected incidence of tandem dinucleotide substitutions in healthy donors. **(A)** Dinucleotide substitutions from unique IGHV, IGKV and IGLV sequences from the Leiden healthy donor cohort and corrected after *in silico* predictions of dinucleotide substitutions that did not occur in tandem. Burgundy cells represent sequence inversions, light and dark purple cells represent juxtalocations of the 5’ and 3’ base in the pair (as seen from the non-transcribed strand), respectively. For unshaded cells, juxtalocation could not be assessed due to one or more nucleotides in the reference sequence matching the mutated sequence. **(B)** Relative contribution of sequence inversions and juxtalocations.

After subtraction of *in silico* predicted ‘false tandem’ TDNS, substitution tables were generated for IGHV, IGKV and IGLV collections and for the complete dataset (IGV **Figure 4**; IGHV, IGKV and IGLV **Supplementary Figure S4**; **Supplementary Tables S7**-**S9** and **Supplemental Data 1.1–1.23**). Heavy and light chain substitution tables were comparable, except for CT to TA and GA to AG TDNS, which were more prevalent in IGLV, and IGKV+IGLV, respectively. We postulate that these observations stem from differences in the reference sequences, in which these bases may be more prone to mutation, or such mutations are more permissible and lead to less negative selection.

TDNS did not occur in equal frequencies, nor did they follow substitution patterns expected from SNS tables. Instead, half (49,9%) of all TDNS occurred in dipurine or dipyrimidine motifs and virtually all mutations swapped the positions of either one of the reference bases (i.e. AG to CA; 51,5% versus 44,4% expected; Student’s t-test: p<0,0001), or both bases at once, leading to inversions (i.e. AG to GA; 37.3% versus 11.1% expected; Student’s t-test: p<0,0001). Only 10,4% (expected: 44,4%; Student’s t-test: p<0,0001) of TDNS did not follow this pattern (**Figure 4** and **Supplementary Table S10A**).

Additionally, we compared the TDNS signature with the recently described doublet base substitutions (DBS) signatures from COSMIC (v3.1 - May 2020). We found that the tandem substitution signature in immunoglobulins does not correspond to a previously described pattern (cosine similarity < 0,5, **Supplementary Table S11**).

Whenever a TDNS reference sequence was frequently found in the TDNS table, their reverse complement would also be highly prevalent. However, within such pairs, the motifs that contained a cytosine on the transcribed/non-coding strand, which during replication serves as a template for lagging strand synthesis, were more frequently mutated than their non-transcribed partner, following the pattern previously described for AID-induced mutations (53) (**Supplementary Figure S5**). Indeed, a strand-bias analysis revealed that tandem substitutions preferentially occurred on the template strand, and that canonical (WRCY) and non-canonical (WA; POLH-associated) (43, 54) AID motifs were most commonly found around the 5’ base of the template strand sequence (**Figure 3**). These observations implicate that tandem substitutions preferentially arise at AID-induced DNA lesions.

**Figure 5:**
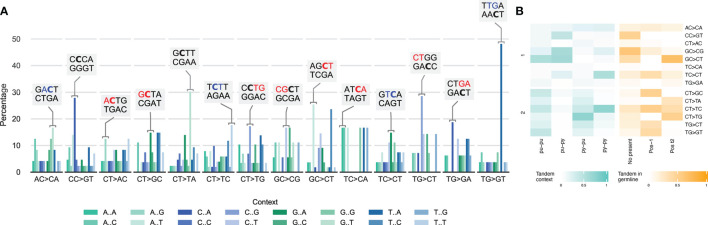
Nucleotide context of tandem dinucleotide substitutions. **(A)** The 14 most frequently found tandem dinucleotide substitutions (and its reverse complement) in IGHV from the Leiden healthy donor cohort, were grouped by all 16 possible combinations of flanking nucleotides. Labels displaying the nucleotide context (and reverse complement) with the highest incidence, in red is highlighted the presence of the tandem in the germline reference and underlined and bold the cytosine in the tandem or nearer to the tandem. **(B)** Tandem germline context analysis (left side, green heatmap). It was performed this analysis considering the base type (py: pyrimidine and pu: purine) using 1 nucleotide before and after the tandem position (the corresponding reference is represented with “-”). Tandem germline context analysis for IGHV sequences (right side, orange heatmap). The presence of the resulting tandem in the germline context was identified +/-1 nucleotide for the 14 more prevalent tandems (No present: tandem was not found in 4 nucleotide context; Pos -1: tandem was found in position -1 considering the tandem as central positions t1t2; Pos t2: tandem was found in position t2 of the tandem).

To identify a common mutational signature, we mapped the nucleotides directly adjacent to tandem substitutions. Scrutiny of these contexts revealed that tandem substitutions commonly already contained the mutated sequence in the overlap between the reference sequence and its context (**Figure 5**). In other words, tandem substitutions apparently derive from small juxtalocation events, where directly adjacent sequences move a single position upstream or downstream, thus explaining the abovementioned observation of one of the original bases moving to the other position in the motif. This mechanism also explains the high incidence of inverting substitutions, as they may result from both upstream and downstream juxtalocations. Although, not found in sufficient abundance to allow for statistical testing, it appeared that tandem substitutions longer than TDNS followed similar patterns.

### Polydipyrimidine Stretches Are Favored

Following the observations that TDNS occur preferentially in dipyrimidine motifs, that the cytosine-containing strand is dominantly targeted, and that tandem substitutions in majority represent single nucleotide juxtapositions, tandem substitutions should preferentially arise from polydipyrimidine stretches. Indeed, the substitution tables show that most of the dominantly observed TDNS motifs fit within this hypothesis (**Figure 4** and **Supplementary Table S10**).

Notable exceptions to this rule were the commonly observed AG to GA and GC to CG inverting substitutions, which contained the mutated sequence in their germline context in less than half of instances.

### VDJ Tandem Substitutions in UNG and MMR Deficiency

After AID deaminates a C into a U, immunoglobulin gene mutational patterns are governed by UNG-intiated BER and the non-canonical MMR pathway. Based on knockout mouse models, tandem substitutions were described to predominantly occur in the MMR pathway (55). Since experimental models to mimic SHM in humans are not available, the closest approximation to test the relative contribution of BER and MMR is to analyze BCR repertoires from DNA repair deficient patients.

We obtained a massive parallel sequencing library with VDJ from one MSH2-deficient and three MSH6-deficient patients. All patients carried biallelic defects in their respective genes, leading to Constitutional MisMatch Repair Deficiency (CMMRD) syndrome (56, 57). An additional VDJ library was obtained from an UNG-deficient patient. As internal controls, we analyzed an independent cohort of healthy donors generated by the same methodology as these patient datasets (henceforth referred to as: ‘Rotterdam healthy donor cohort) (49). Sequences were filtered identically as described for the Leiden healthy donor cohort, except for the selection by mutation load (see *Materials and Methods*), yielding 7.654 unique VDJ sequences from the CMMRD patients and 104 unique VDJ sequences from the UNG-deficient patient.

The CMMRD patient repertoires mostly lacked IgM and both the patient and Rotterdam healthy donor repertoires were sequenced using a different approach and sequencing platform than the Leiden healthy donor cohort, which resulted in higher mutation loads and relatively more false TDNS. Therefore, we did not pool these repertoires in our *in silico* mutation algorithm, but processed them separately and then independently predicted SNS mutation clusters as described for the Leiden cohort.

After *in silico* correction, tandem dinucleotide substitutions in the Rotterdam healthy donor cohort confirmed the previously observed paradigm of juxtapositions in TCT-motifs (**Supplementary Figure S6**, **Supplementary Table S10B**, **Supplemental Data 2.1-2.42)**. In this dataset, inversions and base swaps were less dominant in motifs other than the dipyramidine ones, putatively as a result of the more challenging *in silico* correction of this more artefact-prone dataset compared to the Leiden healthy donor cohort. Nevertheless, in the corrected tables, TDNS still represented 1,9% of all substitutions, almost twice the frequency of the previously highest estimate.

**Figure 6:**
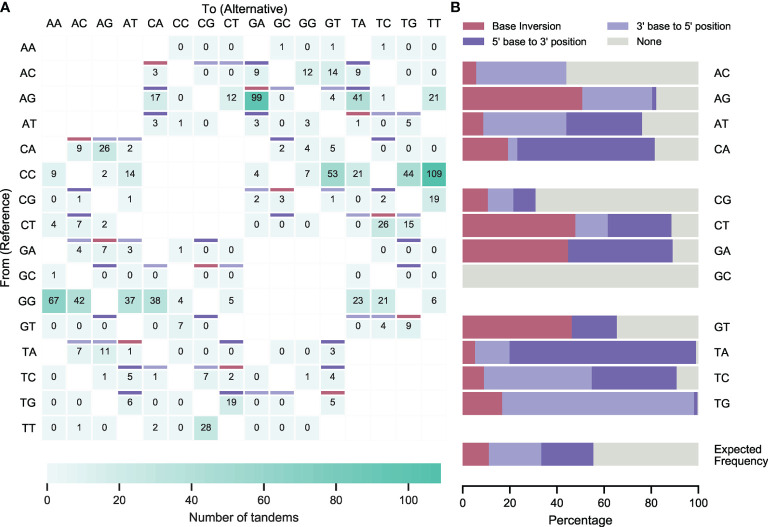
Corrected incidence of tandem dinucleotide substitutions in MSH2/6 deficiency. **(A)** Dinucleotide substitutions from unique IGHV sequences obtained from constitutional mismatch repair deficiency (CMMRD) patients and corrected after *in silico* predictions of dinucleotide substitutions that did not occur in tandem. Burgundy cells represent sequence inversions, light and dark purple cells represent juxtalocations of the 5’ and 3’ base in the pair (as seen from the non-transcribed strand), respectively. For unshaded cells, juxtalocation could not be assessed due to one or more nucleotides in the reference sequence matching the mutated sequence. **(B)** Relative contribution of sequence inversions and juxtalocations.

After removal of *in silico* predicted ‘false’ TDNS, the dinucleotide substitution table of the CMMRD patients also showed great similarities to the Leiden healthy donor cohort, suggesting that the mismatch repair machinery does not play a major role in the formation of tandem substitutions in humans (**Figure 6**, **Supplementary Table S10C**, **Supplemental Data 2.1-2.32**). The corrected proportion of TDNS in all substitutions was 2,0%, comparable to the 1,9% in the equally extensively mutated sequences from the Rotterdam healthy donor cohort. Notably, the SNV show a moderately increased Ts : Tv ratio of 1,91, consistent with DNA repair deficiency during somatic hypermutation (**Supplementary Table S5B**).

**Figure 7:**
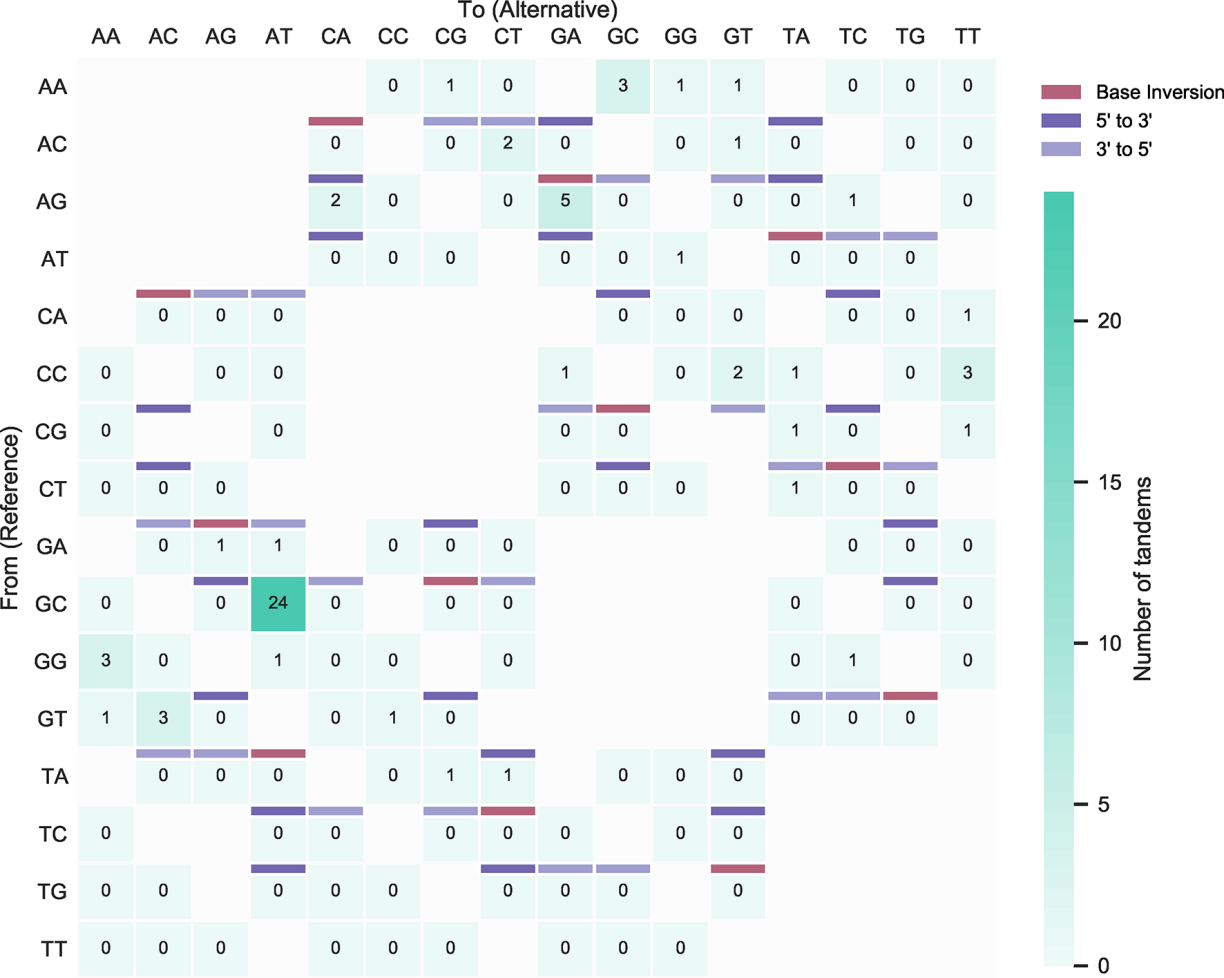
Incidence of tandem dinucleotide substitutions in UNG deficiency. All dinucleotide substitutions from unique IGHV sequences obtained from an UNG deficient patient. Burgundy cells represent sequence inversions, light and dark purple cells represent juxtalocations of the 5’ and 3’ base in the pair (as seen from the non-transcribed strand), respectively. For unshaded cells, juxtalocation could not be assessed due to one or more nucleotides in the reference sequence matching the mutated sequence. Results could not be reliably corrected for dinucleotide substitutions that did not occur in tandem for each mutation independently due to the size of the dataset. However, simulation of dinucleotide substitutions resulting from independent single nucleotide substitutions showed an almost identical number of dinucleotide substitutions (n=76) as the number that was observed (n=67), suggesting that this UNG deficient patient has no tandem substitution events.

Conversely, the previously identified substitution patterns could not be reproduced in the dinucleotide substitution table of the UNG-deficient patient. In the SNS, the Ts : Tv ratio was skewed towards transitions (3,84), consistent with the ‘replication without repair’ pathway leading to C to T transitions in the absence of BER (**Supplementary Table S5C**). Rather than base swaps and inversions, contiguous substitutions dominantly also resulted from this ‘replication without repair’ pathway, representing AID-induced C to T transitions in dinucleotide motifs consisting solely of cytosine and guanine bases (**Figure 7**). This observation remained consistently different from the other datasets when these were randomly sampled down to the smaller library size of this (**Supplementary Figure S7**). Indeed, 81% of these ubiquitous GC to AT transitions were located in AID hotspots, which are known to favor transversions only with UNG (over)expression (58). The paucity of unique sequences in this library precluded detailed *in silico* correction as performed for the other datasets. To obtain the best approximation of the incidence of corrected tandem substitutions in this dataset, *in silico* predictions were performed on pooled sequences of the IGHV3 and IGHV4 families, respectively. These analyses predicted 76 mutation clusters resulting from adjacent SNS events, of which 67 were actually observed in the dataset (**Figure 1** and **Supplemental Data 4.1-4.3**). Thus, no tandem substitutions were observed in the absence of the critical, BER-initiating component UNG. Indeed, this is in line with our previous observation that tandem substitutions are more abundant in immunoglobulin loci than the genome at large, as UNG activity is mainly directed to these genetic regions. Additionally, relative absence of faithful DNA repair mechanisms during SHM makes UNG-derived AP sites more vulnerable to tandem substitutions than genome-wide events of spontaneous deamination (59).

While these findings are congruent with the hypothesis that UNG may be involved in the formation of the majority of tandem substitutions in humans, it should be noted that the n=1 sample size precludes any definitive conclusions.

## Discussion

We describe the ubiquitous presence of tandem substitutions in human V(D)J rearrangements. Tandem substitutions contribute to the acquisition of mutations during SHM in a frequency at least two to five times higher than the previously highest estimate. In fact, tandem substitution events are likely to be still underestimated by this study, since any TDNS events resulting in a mutation where one of the bases matches the reference sequence would appear as a regular SNS. Tandem substitutions have the potential to expedite the adaptive immune response by overcoming amino acid code redundancy and by incidentally mutating multiple residues at once. Clustering of such mutations around AID hotspots and their overall distribution indicates that tandem substitutions are an integral part of the SHM spectrum.

Virtually all tandem mutations adhere to a previously unrecognised substitution pattern resulting from single nucleotide juxtalocations. Confirmation of these findings in the independent Rotterdam cohort generated by a different methodical approach, albeit at a lower frequency, ensures that our observations are not a result of methodological artefacts. As an explanation for this phenomenon, we propose the EXPEDITE (EXtruded Pinching Effecting DIrectional Tandem Exchange) model. In this model, an abasic site transiently adopts an extruded configuration leading to misreading of template DNA during replication. Our data indicate that AID-mediated deamination of a cytosine in polydypirimidine stretches is the main route to the juxtalocations that underlie tandem substitutions. After recognition of the uracil, UNG cleaves the N-glycosylic bond to create an apyrimidinic (AP) site, a known risk for strand slippage (60). Additionally, UNG creates ~45° kinks at dUTP positions (61), which facilitate the extrusion and subsequent skipping of the abasic site by a DNA damage tolerant DNA polymerase. Considering the polydypirimidine motifs, such extruded positioning could be facilitated by the relatively small flanking pyrimidine bases, in a similar fashion as was recently described for flanking cytosine bases (62). Indeed, mismatched pairs have an increased propensity for spontaneous base flipping (63), and the presence of UNG further increases the lifetime of open states following spontaneous base-pair dissociation and twists the unstacked nucleotides out of their helical conformation (64–66). These mechanisms might be considered intrinsic to the canonical BER pathway, but lead to a previously unrecognized effect.

**Figure 8:**
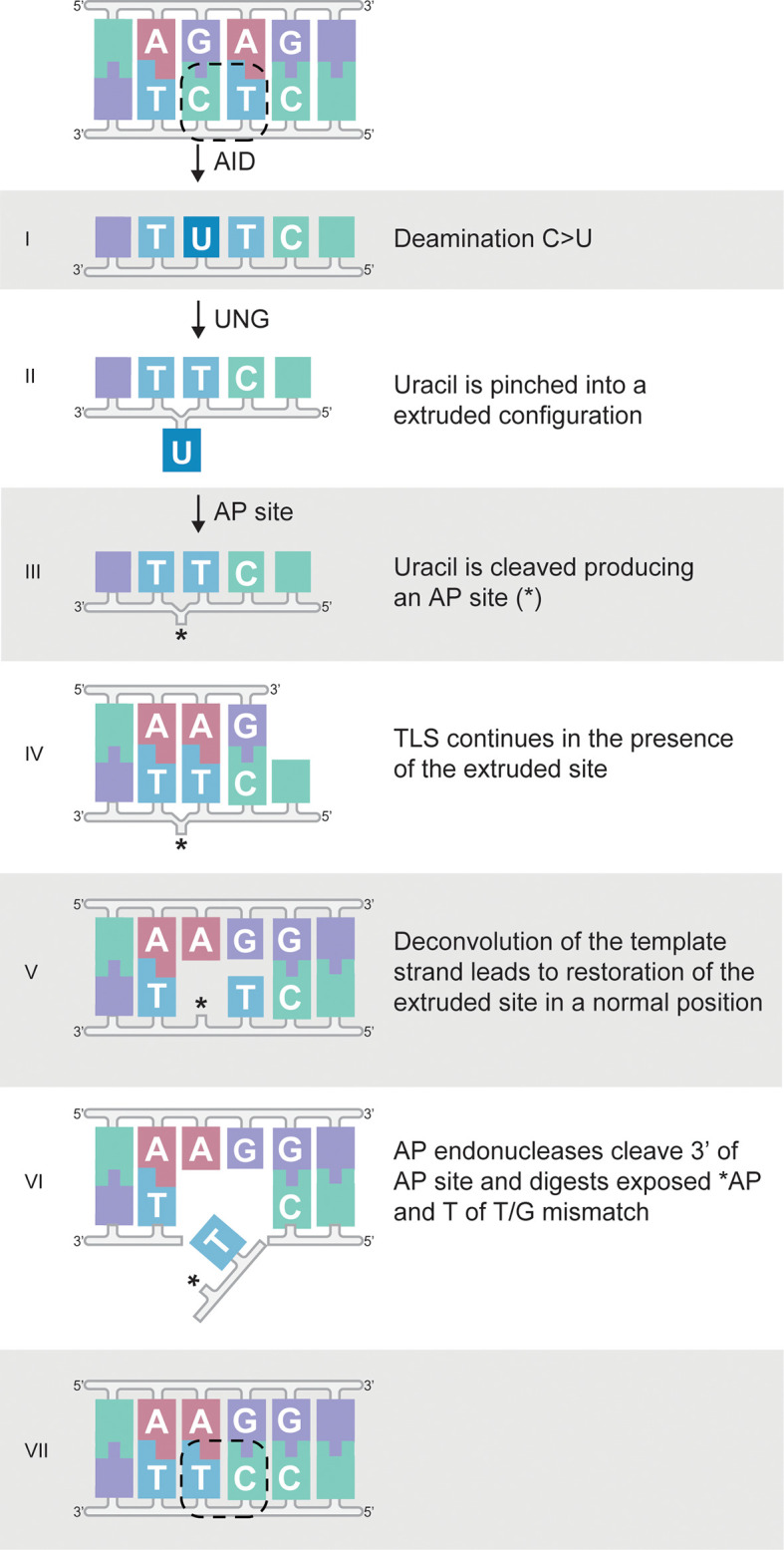
Proposed EXPEDITE model for tandem substitutions causing juxtalocation of adjacent residues during DNA replication. (I) Cytosine (C) is deaminated to uracil (U) in a polypyrimidine context (shown here as an example since this context typically contains the most tandem substitutions, refer to ). (II) UNG pinches the U into an extruded configuration and (III) U is cleaved, leaving an AP site (*). (IV) If during DNA replication the resulting extruded AP site is tolerated, the juxtaposed base acts transiently as a substitute template. (V) After decontraction of the template strand, the AP site is reverted to a normal configuration and replication continues from the ‘mismatch’ position containing the AP site. (VI) Hereafter, the AP site, as well as the mismatch, is removed by canonical base excision repair. (see *Discussion*). (VII) Final repair synthesis and ligation.

Subsequent repositioning of the pinched or extruded AP site or base into the strand backbone would move the sequence one base upstream. Replication by a translesion synthesis DNA polymerase capable of extending from the mismatch ensures further extension of the novel DNA strand. Although the events in this model now no longer follow the canonical BER pathway, recruitment of an AP endonuclease (APE), as a downstream component of BER, is conceivable. APE cleaves 3’ of the AP site and removes the adjacent mismatched base. Finally, the newly generated gap in the template strand is filled and ligated by faithful DNA repair mechanisms, thereby completing the TDNS process (**Figure 8**). The length of the gap, minus any chance matches at its termini, determines the length of the tandem substitution. It should be stressed that the EXPEDITE model is a best estimate proposal, and the exact proteins and mechanisms involved, especially in the non-canonical mechanism following UNG involvement, are subject to speculation and await verification.

Although AP sites, mismatched bases, and uracil residues have a particular proclivity to adopt extruded configurations (62, 63, 67–69), juxtalocations could theoretically also result from spontaneous base flipping of matched base pairs, especially A-T pairs (66). Indeed, we observe that germline motifs AT and TA, which contain no cytosine bases on either strand and would therefore remain unexplained through the UNG pathway, are the least abundant reference motifs of all tandem substitutions - yet are not absent. Furthermore, they do follow the paradigm of single nucleotide juxtalocated outcomes. Alternatively, these juxtalocations may be part of a longer tandem substitution initiated by a cytosine that is near, but not inside the motif, and other bases in this longer tandem substitution remain identical after juxtalocation, therefore not registering as mutated.

Not all tandem substitutions follow the juxtalocation paradigm. Most importantly, the common (top strand) AG to GA inverting substitution contained the mutated sequence and their direct context in only less than half of cases, suggesting that these may stem from an alternative mechanism. We consider the following: deamination of the C in the bottom strand leads to U, and subsequent replication across U results in a G to A mutation on the top strand (canonical C to T on the bottom strand). Following removal of the U on the bottom strand by either canonical UNG or canonical MMR and patch repair of the bottom strand by POLH, replication across from the new WA (POLH hotspot) introduces a C across from the A. This will generate the observed AG to GA substitution (**Supplementary Figure S8**). Thus it appears that similar to previous observations in mice, MMR is to some extent also involved in tandem substitution generation in humans. However, in contrast to murine studies (42), a majority of tandem substitutions in humans appear to depend on UNG activity in the BER pathway, marking a notable species difference.

A number of specific TDNS, most importantly GC to AA/TT and TC to AA, have previously been attributed to POLZ and/or POLI activity following observations in *Saccharomyces cerevisiae* and murine models (35, 42, 70, 71). However, these TDNS are among the rarest in each of our datasets and therefore, at least in humans, do not seem to represent significant additional pathways beyond the mechanisms described in this manuscript. Supposedly, these differences derive from a previously remarked species difference concerning tandem substitution formation (35). Indeed, murine translesion polymerases create tandem substitutions through the MMR pathway (42), whilst experiments in human cell lines have implicated POLI in tandem substitution formation, but rather through the involvement of UNG (72, 73). The data in this report propose a new model where the actual tandem substitution inducing lesion, the AP site, does not serve as a non-instructive template but rather causes juxtapositioning by template flipping. Several characteristics identify POLH as the prime candidate translesion synthesis polymerase to create tandem substitution during SHM: The requirement of a large catalytic site, the open active POLH site that can accommodate non-Watson-Crick base pairs, and the high POLH error rate of 3,5x10^-2^ which is associated with its ability to bind dNTP without DNA substrate (74, 75).

All observations in this study derive from a reverse immunology approach. The lack of experimental systems to study SHM in humans currently precludes controlled experimental confirmation of our proposed mechanism. Therefore, analysis of gene-deficient patients as performed in this study serves as a preliminary attempt of experimental confirmation. When testing the EXPEDITE hypothesis against a small cohort of DNA repair deficient patients, we found an apparent absence of tandem substitutions in UNG but not in MSH2/6 deficiency. Although these findings corroborate the described model with a strict dependence on abasic sites, the small number of individuals and sequences and the more error-prone sequencing approach preclude any definitive confirmation. Therefore, the analysis of additional UNG-deficient patients with high-fidelity BCR amplification and sequencing would be highly desirable. Unfortunately, homozygous UNG deficiency is an exceptionally rare disease (76), and the national reference centers for immunodeficiency of The Netherlands (Erasmus Medical Center Rotterdam), France (Hôpital Necker-Enfants Malades, Paris), and Germany (University Medical Center Freiburg) do not have additional UNG-deficient patients in their registries and were also unable to provide us material from the three originally described patients (77). We recommend that whenever additional research material comes available, additional B-cell receptor repertoires are sequenced and results are shared with the scientific community to elucidate the exact tole of UNG in the formation of tandem substitutions.

## Data Availability Statement

All the other data supporting the findings of this study are available within the article and in the repository: https://catg.cl/lab/our-articles/jsepulveda-tandem-substitutions/. The code that was written for this project is publicly available at: https://github.com/catg-umag/tandem-substitution-simulation.

## Ethics Statement

The studies involving human participants were reviewed and approved by the local ethics committee (no. B16.039), Leiden University Medical Center. Written informed consent to participate in this study was provided by the participants’ legal guardian/next of kin.

## Author Contributions

JS-Y and MK designed the study. MK performed experiments. JS-Y and MK performed analyses. DA-S and MN performed bioinformatics experiments and analysis. JS-Y, BP, HJ, and MK proposed the model. HV, MB, MN, and HJ advised on the project. MK and HJ wrote the manuscript. All authors have read and approved the manuscript.

## Funding

MK was supported by a grant from the Professor Steenhuis Fonds. HJ received TOP Grant [91213018] from the Dutch Scientific Organization NWO-ZonMW. JS-Y received a Doctorado Becas Chile [2016-72170683] from Agency for Research and Development (ANID). MN and DA-S work was supported by Fondo Nacional de Desarrollo Científico y Tecnológico (Fondecyt) [1180882], [11140542], and [MAG1895].

## Conflict of Interest

The authors declare that the research was conducted in the absence of any commercial or financial relationships that could be construed as a potential conflict of interest.

## Publisher’s Note

All claims expressed in this article are solely those of the authors and do not necessarily represent those of their affiliated organizations, or those of the publisher, the editors and the reviewers. Any product that may be evaluated in this article, or claim that may be made by its manufacturer, is not guaranteed or endorsed by the publisher.
