# Supplementary material for: Tandem Substitutions in Somatic Hypermutation
Source: Front Immunol. 2022 Jan 7;12:807015. doi: 10.3389/fimmu.2021.807015 (PMC8781386; doi:10.3389/fimmu.2021.807015)
Supplement: Supplementary file 2 [file DataSheet_1.pdf]

Supplemental material

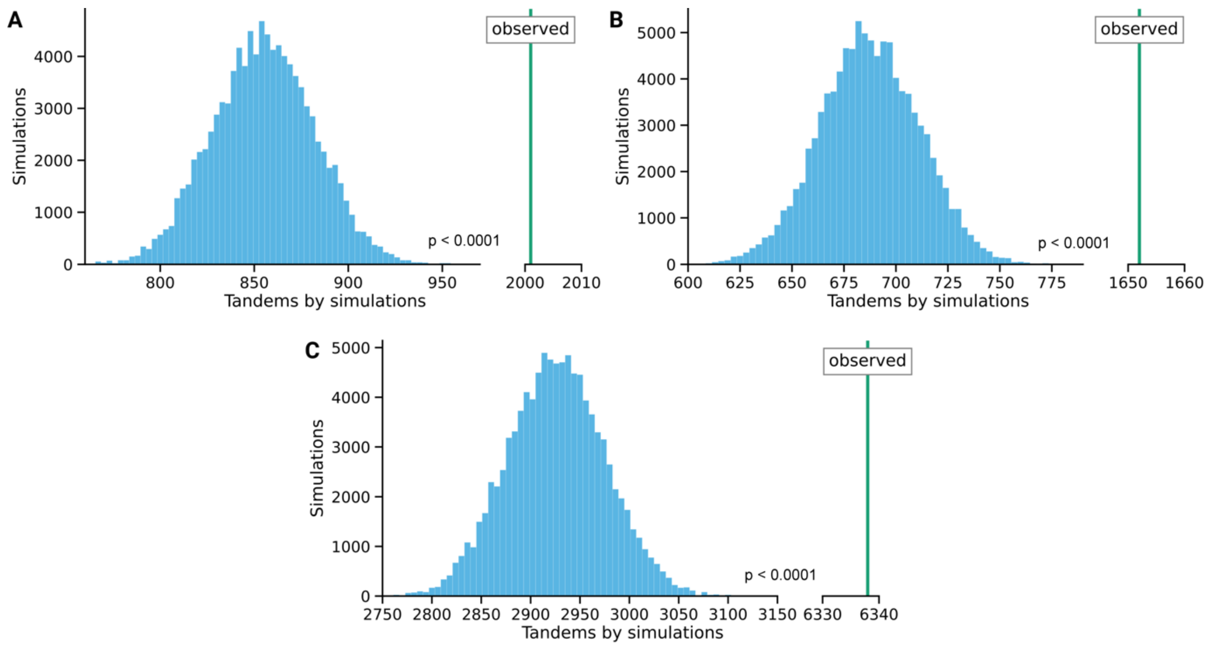
A
observed
Simulations
4000
3000
2000
1000
0
p < 0.0001
800
850
900
950
2000
2010
Tandems by simulations
B
observed
Simulations
5000
4000
3000
2000
1000
0
p < 0.0001
600
625
650
675
700
725
750
775
1650
1660
Tandems by simulations
C
observed
Simulations
5000
4000
3000
2000
1000
0
p < 0.0001
2750
2800
2850
2900
2950
3000
3050
3100
3150
6330
6340
Tandems by simulations

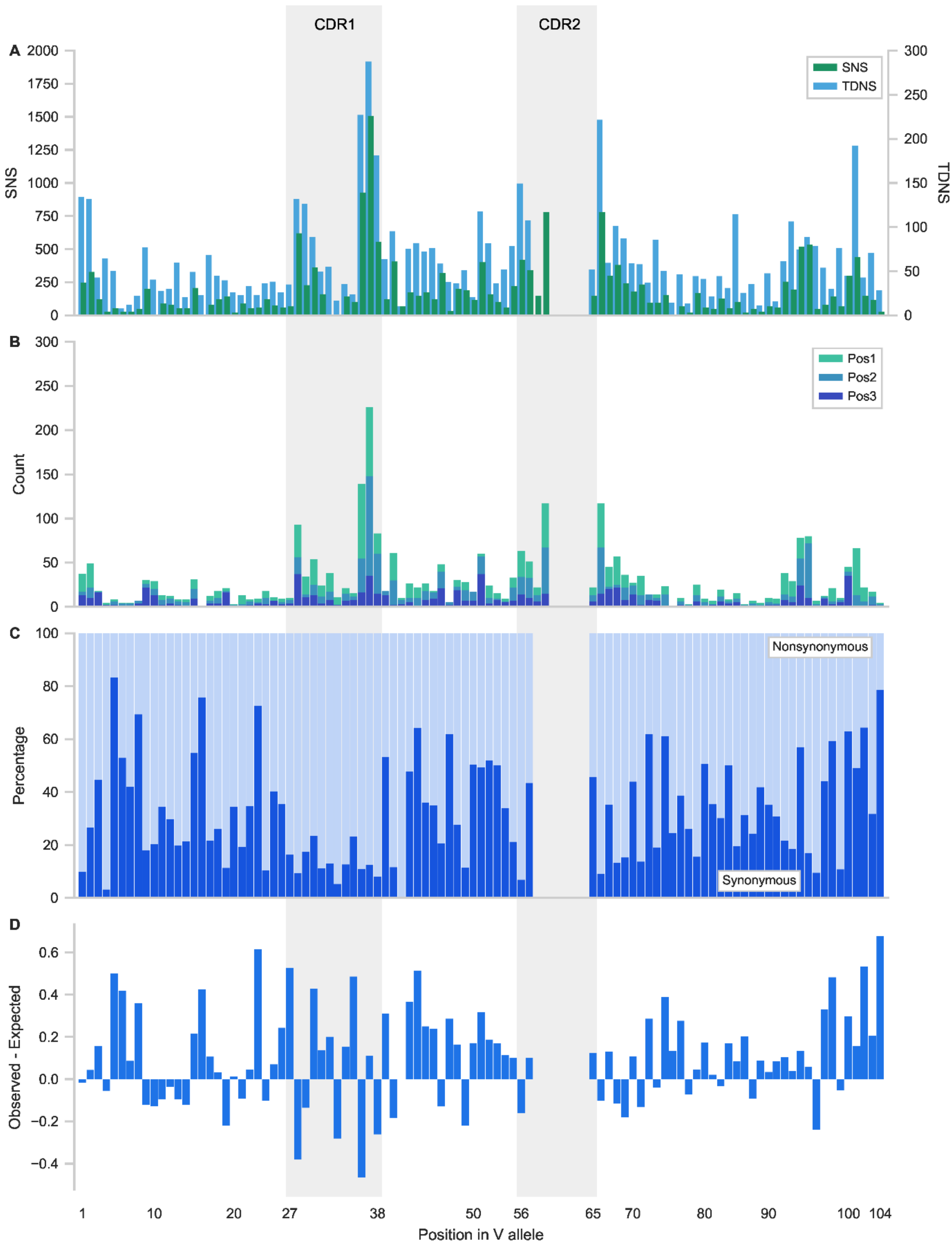

Supplementary Figure S2. **Distribution of tandem dinucleotide substitutions in the IGKV allele. (A)** Distribution of single nucleotide substitutions (SNS) and tandem dinucleotide substitutions (TDNS) in the IGKV allele. **(B)** Nucleotide specific context within a codon. Relative contributions of tandem dinucleotide substitutions for VDJ. Results are split per position in the codon. Position 1 represents 5' and middle base on the coding strand, position 2 represents middle and 3' base and position 3 represents the 3' base of the codon and the 5' base of the downstream codon. **(C)** Proportion of synonymous and nonsynonymous mutations per position

in the IGKV allele. **(D)** Mutational resistance score. Plotted are the expected minus the observed frequencies of nonsynonymous mutations per codon position in the IGKV allele. Increasingly higher values represent residues that are more resistant to mutation.

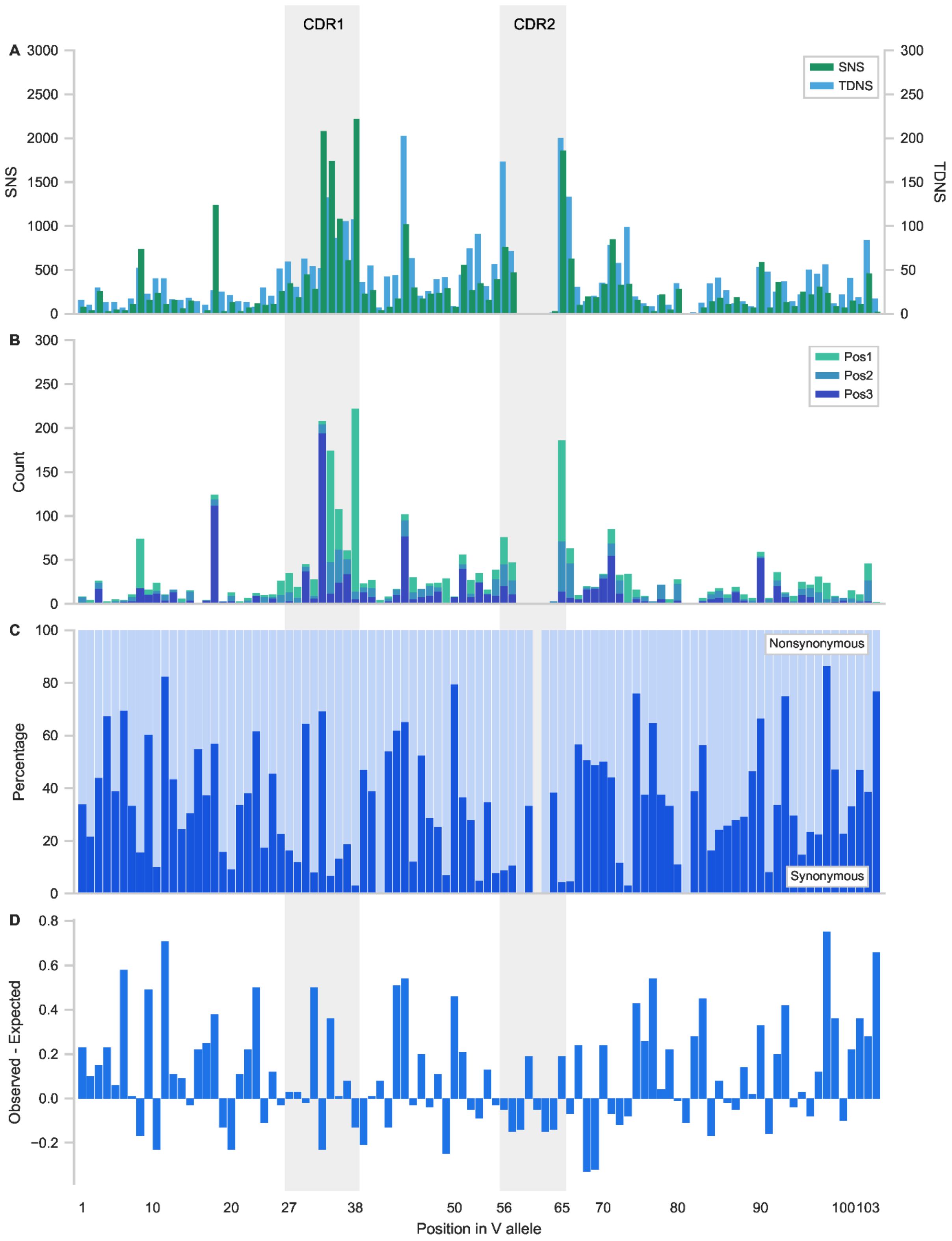

Supplementary Figure S3. **Distribution of tandem dinucleotide substitutions in the IGLV allele. (A)** Distribution of single nucleotide substitutions (SNS) and tandem dinucleotide substitutions (TDNS) in the IGLV allele. **(B)** Nucleotide specific context within a codon. Relative contributions of tandem dinucleotide substitutions for VDJ. Results are split per position in the codon. Position 1 represents 5’ and middle base on the coding strand, position 2 represents middle and 3’ base and position 3 represents the 3’ base of the codon and the 5’ base of the downstream codon. **(C)** Proportion of synonymous and nonsynonymous mutations per position

in the IGLV allele. **(D)** Mutational resistance score. Plotted are the expected minus the observed frequencies of nonsynonymous mutations per codon position in the IGLV allele. Increasingly higher values represent residues that are more resistant to mutation.

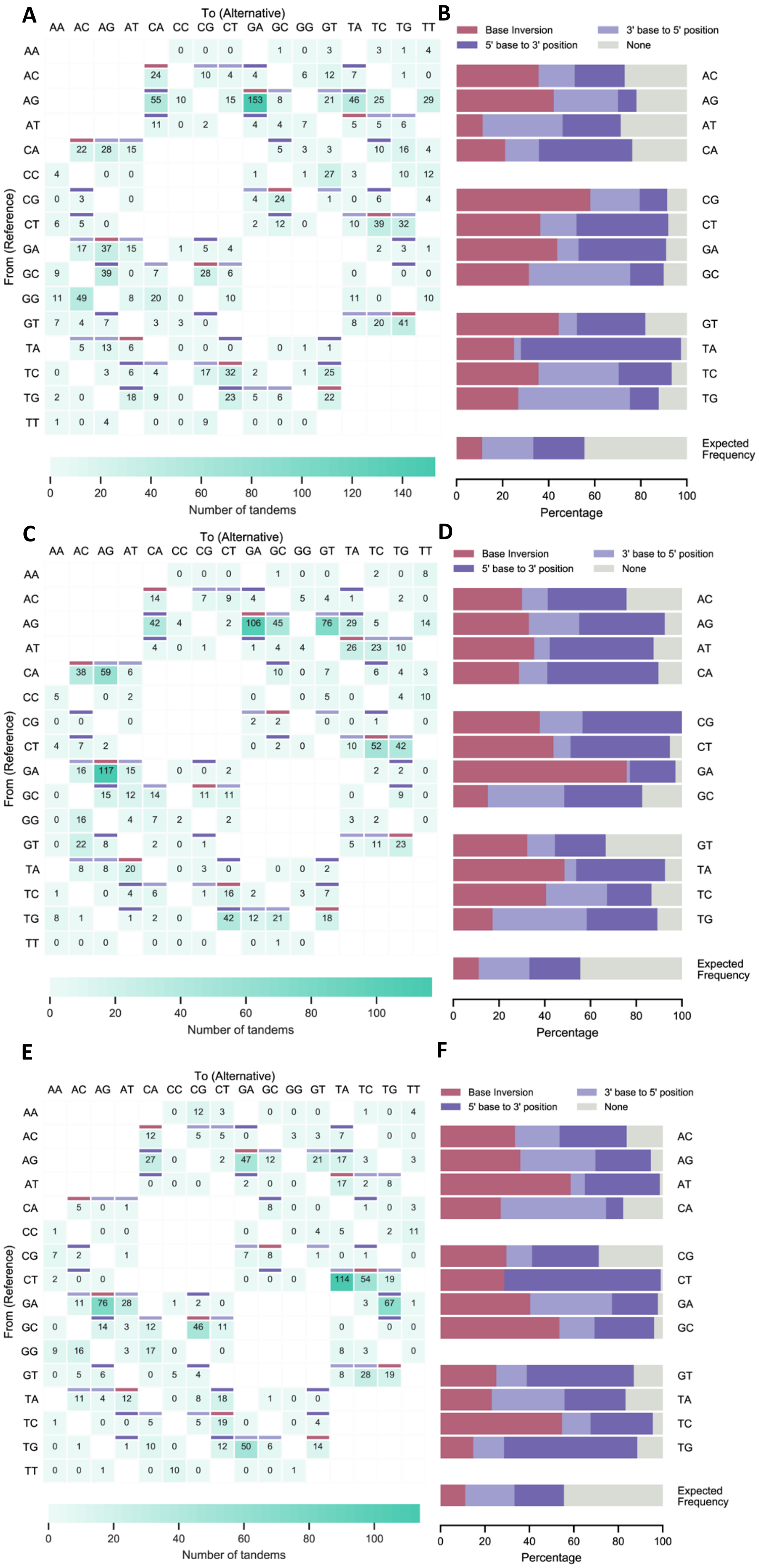
A
To (Alternative)
From (Reference)
AA AC AG AT CA CC CG CT GA GC GG GT TA TC TG TT
Number of tandems
B
Base Inversion
5' base to 3' position
3' base to 5' position
None
Expected Frequency
Percentage
C
To (Alternative)
From (Reference)
Number of tandems
D
Base Inversion
5' base to 3' position
3' base to 5' position
None
Expected Frequency
Percentage
E
To (Alternative)
From (Reference)
Number of tandems
F
Base Inversion
5' base to 3' position
3' base to 5' position
None
Expected Frequency
Percentage

Supplementary Figure S4. **Corrected incidence of dinucleotide tandem substitutions in Leiden healthy donors by chain. (A)** Data obtained from unique IGHV sequences and corrected after in silico predictions of 'false' tandems. **(B)** Relative contribution of inversions and position changes. **(C)** Data obtained from unique IGKV sequences and corrected after in silico predictions of 'false' tandems. **(D)** Relative contribution of inversions and position changes. **(E)** Data obtained from unique IGLV sequences and corrected after in silico predictions of 'false' tandems. **(F)** Relative contribution of inversions and position changes. Granate cells represent sequence inversions, light and dark purple cells represent juxtalocations of the 5' and 3' base in the pair (as seen from the non-transcribed strand), respectively. For grey shaded numbers, juxtalocation could not be assessed due to the reference sequence consisting of two identical nucleotides.

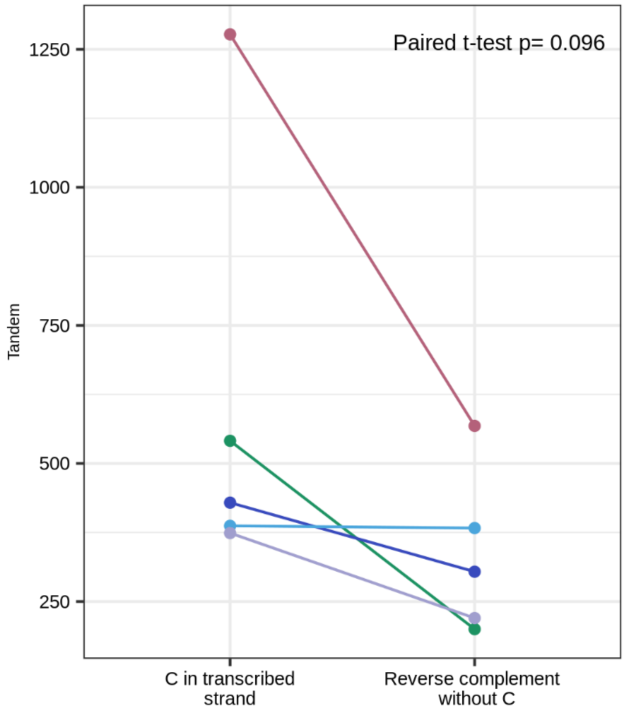

Supplementary Figure S5. **Strand bias in tandem substitutions.** Paired dinucleotide motifs with their reverse complementary counterparts, showing that sequences with a cytosine on the transcribed strand (represented as a guanine on the coding strand motif) occur more frequently than sequences with the same motif on the non-transcribed strand.

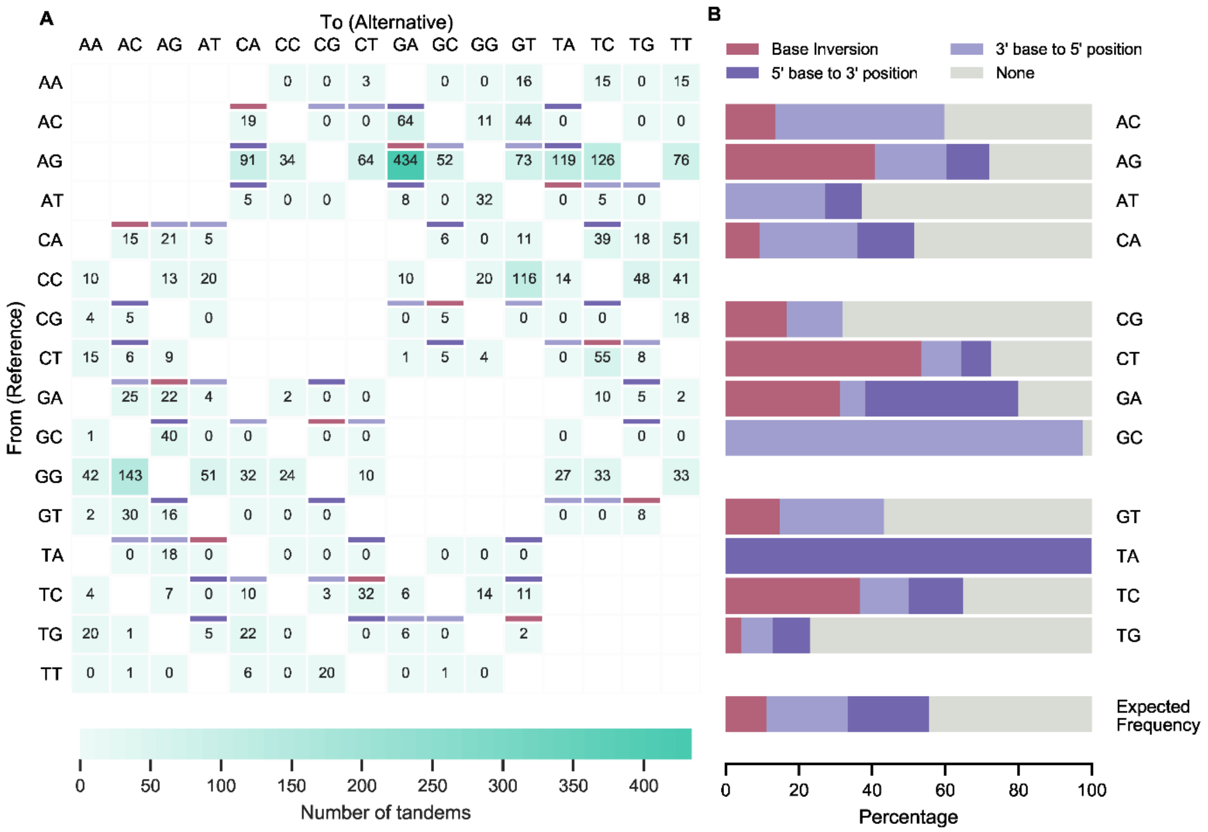

Supplementary Figure S6. **Corrected incidence of dinucleotide tandem substitutions. (A)** Data obtained from unique IGHV sequences from the Rotterdam healthy donor cohort and and corrected after in silico predictions of 'false' tandems. Granate cells represent sequence inversions, light and dark purple cells represent juxtalocations of the 5' and 3' base in the pair (as seen from the non-transcribed strand), respectively. For grey shaded numbers, juxtalocation could not be assessed due to the reference sequence consisting of two identical nucleotides. **(B)** Relative contribution of inversions and position changes.

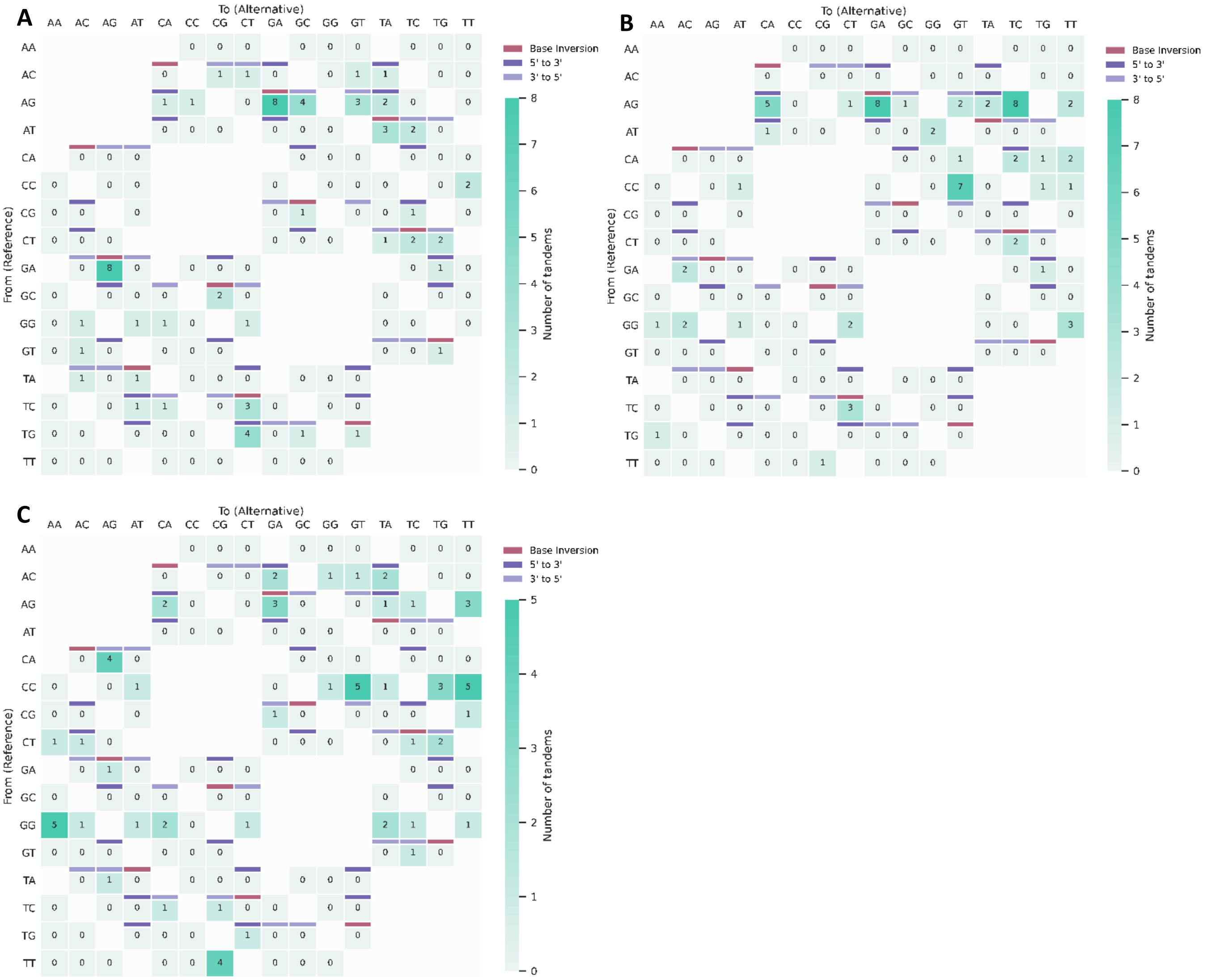

Supplementary Figure S7. **Incidence of dinucleotide tandem substitutions in a random subset (normalized according to UNG-deficient library) of Leiden healthy donors, Rotterdam healthy donor and MSH2/6 deficiency cohort. (A)** From the Leiden healthy donor cohort, a random subset was sampled to the size of the UNG-deficient library. **(B)** From the Rotterdam healthy donor cohort, a random subset was sampled to the size of the UNG-deficient library. **(C)** From the MSH2/6-deficient cohort, a random subset was sampled to the size of the UNG-deficient library. Granate cells represent sequence inversions, light and dark purple cells represent juxtalocations of the 5' and 3' base in the pair (as seen from the non-transcribed strand), respectively. For grey shaded numbers, juxtalocation could not be assessed due to the reference sequence consisting of two identical nucleotides.

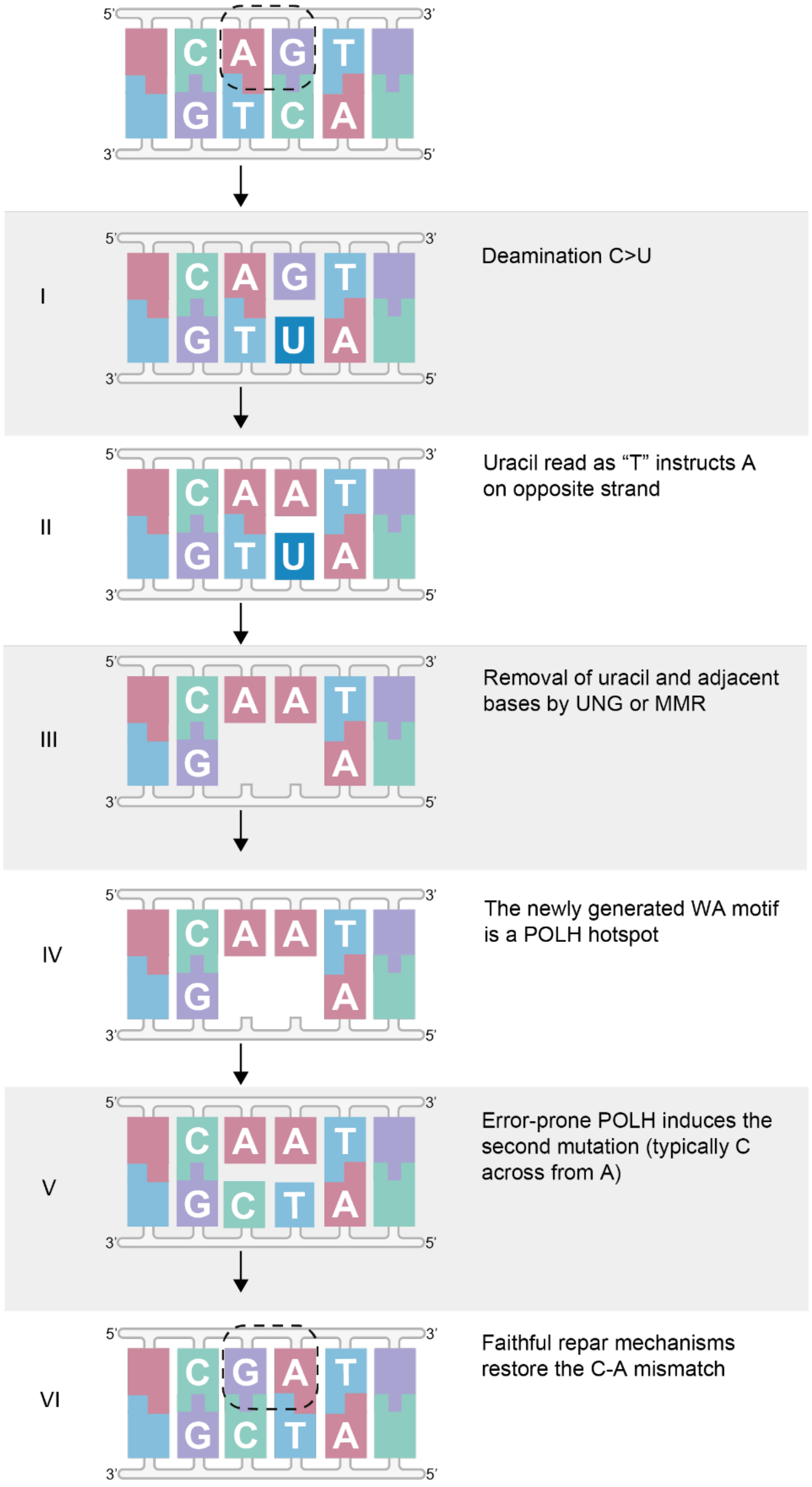

Supplementary Figure S8. **Model for AG>GA tandem substitutions. (I)** Deamination of the C in the bottom strand leads to U. **(II)** Subsequent replication across U results in a G to A mutation on the top strand (canonical C to T on the bottom strand). **(II)** Following removal of the U on the bottom strand by either UNG or MMR. **(IV)** Patch repair of the bottom strand by POLH. **(V)** Replication across from the new WA (POLH hotspot) introduces a C across from the A. **(VI)** This will generate the observed AG to GA substitution.
